# "The Hospital" Nursing Mirror

**Published:** 1898-10-22

**Authors:** 


### "The Hospital" Nursing Mirror.

Being the Nursing Section of "The Hospital."

[Contributions for this Section of "The Hospital" should be addressed to the Editor, The Hospital, 28 & 29, Southampton Street, Strand, London, W.C., and should have the word "Nursing" plainly written in left-hand top corner of the envelope.]

### News from the Nursing World.

### OUR CHRISTMAS DISTRIBUTION.

It is time that our kind friends, who desire that our Christmas distribution of warm and useful gifts to the patients in the London hospitals this year should be a generous one, should set to work in earnest to make it so. The question is not what we want, but what can we do without, so that contributions from those who knit of socks and stockings, mittens and scarfs, cross-overs and caps, and numerous comfortable wraps, are sure to be eagerly looked for; whilst the garments of all sizes and shapes that are turned off so quickly by the ready needlewoman are most welcome. It is piteous to see how short a way a big parcel goes amongst so many, and when our nurses remember that it is upon their sister-nurses in the hospitals and infirmaries that the burden of making Christmas happy for so many falls, they must feel doubly glad to think that their gifts not only comfort the sick, but cheer the hearts of their fellows.

### NURSING AT CARDIFF INFIRMARY.

The value of red tape has rarely been more fully shown than in the report of a meeting of the Cardiff Infirmary Committee last week. One of the subjects under consideration was the resignation of the matron, who gave as a reason for sending it in that things had not been pleasant for her during the last twelve months. Further questioned on the matter, she explained that she considered the friction was occasioned by some irksome bye-rules and by the independent action of individual members of the sub-committee appointed to inquire into the economic administration of the Infirmary. In fact, there has undoubtedly been a misconception on the part of the members of the sub-committee as to the duties attached to the position, and with regard to the proper way of carrying them out, which has caused needless friction. After some discussion, Miss Wilson was asked to continue her work at Cardiff, and we can congratulate her on receiving so encouraging a mark of confidence from the committee.

### ARMY NURSING CORPS FOR NEW SOUTH WALES.

The Government authorities of New South Wales have sanctioned the formation of an "Army Nursing Service Reserve." It will consist of one lady superintendent, one superintendent, and 24 "sisters." Each, in addition to the usual three-year certificate of training from a general hospital, will be required to pass an examination in "first aid," and to attend annually six lectures on the organisation of military hospitals, hygiene, and military surgery. Candidates must be over the age of 21, and under that of 40; and must satisfy the military medical officers of their suitability for the work. An allowance of £2 each will be made towards providing a special uniform, similar in design to that worn by the English military nurses, and £1 will be granted yearly for its replenishing. In the case of war these nurses will be called upon for duty, and will be subject to the Volunteer Act.

### COLONIAL AND FOREIGN NEWS.

It is with great pleasure that we chronicle the presentation to Miss Ada L. Burgin of a testimonial and a silver mounted purse containing £18 18s. by the medical staff of the Woodstock Suburban Hospital, Cape Town, where she has been matron for the last ten years. Miss Burgin's nursing staff have also given her a travelling trunk. Both are tokens of affection and esteem, presented upon her resignation and return to England. We are always glad to have particulars of nursing news from abroad, and especially from our English nurses when personally communicated, as in this instance. If nurses in these other lands remembered how full of interest their novel experiences are to their fellows at home, and also how valuable they are to those who meditate emigrating, they would write more frequently, and, in doing so, they would themselves be brought more into touch with the rest of the nursing world.

### BUSINESS LECTURES AT THE TRAINED NURSES' CLUB.

The first of the lectures on business matters, which are being given at the Trained Nurses' Club, 12, Buckingham Street, Strand, took place on Tuesday afternoon, and was well attended. Miss Somerville is an admirable lecturer, speaking with thorough mastery of her subject, and with such clearness of language that her audience could not fail to understand every point. The procedure at meetings and the duties of a chairman were dealt with on Tuesday. It always seems a difficult matter for the feminine mind to grasp details of business, and the average woman is sublimely ignorant of the difference between propositions, resolutions, amendments, &c., and to all who take interest in public questions, or belong to societies, and frequently attend meetings or committees, the information so clearly given was of great value. Other equally important subjects will be treated in the future lectures, which take place on Tuesday and Thursday afternoons, at half past five. Anyone wishing to make use of this excellent opportunity should apply at once to the Hon. Secretary, Miss Toynbee, at the Club.

### NEW NURSING SCHEME FOR CORK.

An entirely new scheme for nursing the South Infirmary and County Hospital, Cork, has lately occupied the attention of the Governors. The matron, Miss Woodroffe, having resigned, from failing health, the trustees undertake the nursing of the infirmary from the 1st inst. It is suggested that a representative nursing committee be appointed, with a matron to directly control the nursing of the institution and the training school of nurses. She will, in conjunction with the nursing committee, choose the probationers, but will not have the power to reject a candidate. There is also to be a staff superintending nurse, who will be appointed by the committee, to act under the matron for the teaching and supervision of the probationers. The trustees will receive all the fees and keep a separate account of all moneys expended and received by the home, in order that the financial success of the plan may be ascertained. The training will be for twenty-one months in the infirmary and for three months in a fever hospital. The candidates will be required to undergo quarterly examinations; and, after receiving their final certificates, may be engaged in the service of the hospital. The resolve of the Governors of the hospital to establish a training school and nurses' home in connection with the infirmary is excellent, and in accordance with the modern ideas; but it is not difficult to pick holes in the way they think of managing it. In the first place, there are too many masters—trustees, committees, matron, superintendent nurse, &c.—and a probationer has the right of appeal to the nursing committee; and, in the second, the period of training is only for two years. At this early stage of the proposal we will only notice two points, namely, that to make a training school for nurses a success there must be a good matron, and the wings of her authority must not be too closely clipped; secondly, training should be for three years, it being the unanimous opinion of all those engaged in the work of teaching nurses that that time is the minimum in which it can be well done.

### THE MIDDLESBROUGH NURSING ASSOCIATION.

The premises in which the nurses of the Middlesbrough Nursing Association are lodged have been sold, and consequently the committee have had to arrange for a speedy removal. Two alternatives present themselves in such a case—a new home can be either rented or bought. There can be no question of the overwhelming advantages of the latter course, especially when, as in the present instance, there is no suitable vacant house to be let. There is also a sum of £400 from the Mayor's Jubilee Fund available for this purpose, so that it would be a pity to let so favourable an opportunity slip. The honorary treasurer has been promised a donation of £50 from one gentleman if five others will give equal sums, and already two contributions have been forthcoming. Small gifts have been promised as well, so that there is a probability of the scheme being brought to a successful termination.

### NURSING ABROAD.

Many of our nurses write and ask us how they may obtain work abroad. The subject is a difficult one, for it is by no means safe from any point of view for women to leave England, where their welfare is so closely watched, for other places where they will have to encounter many hardships, jealousies, and sometimes actual personal danger. In France, for instance, the law permits an employer to dismiss an employé at a moment's notice, and this applies to nurses. Then there are few nursing homes abroad that care to attract English nurses, and a private nurse must have capital and friends at her back before she can start on her own account. The Holland Institute, now advertising in our columns, is one of the best known agencies; and the Victoria Institute of Trained Nurses at Bournemouth has lately opened a branch at St. Servan as an experiment. A small flat has been taken near the post office, and a senior nurse has been put in charge. If successful, this venture will form the nucleus of a nurses' home to supply the English and American colonies at Dinand, St. Malo, St. Servan, and neighbourhood. Particulars of the Victoria Institute may be obtained from the lady superintendent.

### ACCEPTABLE RECOGNITION.

Our letter-box this week has been so pleasantly full of appreciative thanks from our readers for small services which we have been able to render them that we feel moved to say a word or two on this matter. Correspondents are apt to imagine that the editorial actions are so impersonal that any thanks which would be due from one private individual to another are quite unwelcome. But we assure our readers that this is not so. It is a matter of genuine satisfaction to us when we learn that our efforts to assist have proved successful, or that any comments of ours have given pleasure or proved of use.

### NURSING IN THE HEATHFIELD AND WALDRON DISTRICT.

Miss Taite, Queen's Nurse, has just completed her first year's work in the large, scattered district of Heathfield and Waldron. She has nursed 76 cases, and, further help being needed, the idea of engaging an attendant to assist her, and sit up at nights with patients needing such attention, has been broached. The Heathfield, Waldron, and District Nursing Association, under which she works, spent £101 during the year, and has a balance in hand of £24. The association works on provident lines, and a sum of £35 was raised by members' contributions, fees, and donations.

### SHORT ITEMS.

Under the will of the late Dr. Campbell the Perth Sick Nursing Society has received the sum of £100.—All gifts to the Berwick-on-Tweed Ladies' District Nursing Association are acknowledged monthly in the local paper.—A drawing-room meeting to advocate the cause of the Nurses' Total Abstinence League was held at Nottingham on the 12th inst. The Hon. Mrs. Elliot Yorke presided, and Dr. Annie McCall expressed her opinion that from a hygienic point of view nurses could do their work better whilst abstaining from alcoholic liquors.—The Dolgelly Board of Guardians have, at the suggestion of the Local Government Board, subscribed a fixed sum to the Barmouth and Dolgelley District Nursing Fund. After some debate £1 was voted for this purpose.—At the annual meeting of the Crieff District Nursing Association, over which the Countess of Ancaster presided, it was proposed to hold a fancy fair in August of next year in order to raise a sum of £800 to complete the endowment fund.—The Guardians of Gloucester have received the sanction of the Local Government Board to pay £150 as this year's subscription to the funds of the District Nursing Society.—Lady Boughey opened a sale of work lately in the Town Hall, Newport, on behalf of the Benefit Nursing Association. Various entertainments took place in the Corn Exchange during the afternoon, which were well attended. Everything passed off very successfully.—Henry James Gale and Oscar Harman, of the training ship "Arethusa," have each been presented with the vellum certificate of the Royal Humane Society for saving the life of a child at Greenhithe. Gale received his certificate in the presence of his comrades of the "Arethusa," and Harman, who has entered the navy, before the assembled ship's company.—A harvest thanksgiving service was held recently at the Hospital and Home for Incurable Children, 2, Maida Vale, W. A number of friends were present, and the children greatly enjoyed a pretty and touching service.

### Antiseptics for Nurses.

By a Medical Woman.

### XXI.—DISINFECTION BY HEAT.

Heat is by far the best disinfectant hitherto known for the destruction of all organic matter, but, owing to the high temperature necessary to destroy effectually the germs of disease, it is obvious that it is not applicable in many cases. It is, however, extremely useful to be able to boil for ten minutes any article of clothing, or utensil, or other infected article that will bear it, and feel sure that all germs of disease are thereby destroyed. Of late years much attention has been given to the advantages of heat, both dry and moist, for disinfecting purposes, and many forms of apparatus have been invented for its easy application. Dr. Parsons has made some interesting experiments as to the degree of heat necessary to completely destroy germs, and, as the anthrax bacillus is the most resistant, it was used as the standard. The efficiency or otherwise of the various forms of apparatus which have been constructed for destroying infectious matter generally must depend upon whether the heat generated was sufficient to penetrate thoroughly those articles which are most bulky and non-conducting, whilst at the same time it is essential that it should neither injure nor destroy the most delicate fabrics. Heat may be applied in two ways for disinfecting purposes, viz., either dry or moist, and Dr. Parsons has experimented with both, and with the result that he considers the latter by far the more efficient and satisfactory in almost every respect. The main conclusions to which Dr. Parsons has come with regard to these two forms of heat are as follows: A longer time is required when dry heat is employed to destroy germs—an exposure of one hour at a temperature of 220 deg. F. in the case of dry heat, whereas one of five minutes only was sufficient with steam or boiling water at 212 deg. F. Dry heat penetrates very slowly into bulky and badly-conducting articles of bedding or clothing, such as pillows, or mattresses, or rolls of carpet, and consequently the time usually allowed for the disinfection of such articles is quite inadequate to allow a sufficiently high degree of heat to penetrate to the interior. Steam penetrates far more rapidly and effectually than does dry heat, and its power of penetration may be still further increased by employing it under pressure. When dry heat is used, scorching begins to occur at different temperatures with different materials, white wool being the soonest affected. It is specially apt to occur when heat is in the radiant form. To avoid the risk of scorching, heat should not be allowed to exceed 250 deg. F., and even this temperature is too high for white woollen articles.

Steam disinfection is inapplicable in case of leather, or of articles which will not bear wetting. For articles that will stand it, boiling water may be relied on as an efficient means of disinfection. It is necessary, however, that before boiling the grosser dirt should be removed by a preliminary soaking in cold water, because a high temperature, whether moist or dry, has a tendency to fix organic stains indelibly in textile materials. This should be done before the linen leaves the infected place.

The articles for which disinfection by dry heat or steam is specially applicable are such as will not bear boiling in water, such as bedding, blankets, carpets, and cloth clothes generally.

The most important requisites of a good apparatus for disinfection by heat are that the temperature of the interior shall be uniformly distributed; that it shall be capable of being maintained constant for the time during which the operation extends, and that there shall be some trustworthy indication to the actual temperature of the interior at any given movement. In dry heat apparatus, and especially in those heated by coal or coke, the temperature is neither uniformly distributed nor is it maintained constant during the whole time of disinfection. Also in very few of them did the thermometer with which the apparatus was provided afford a trustworthy indication of the temperature of the interior. In steam apparatus all the above requirements are satisfactorily met, and for this reason, as well as for the greater rapidity and certainty of their action, Dr. Parsons considers that steam apparatus are greatly to be preferred to those in which dry heat is used. To still further illustrate the difference between the efficiency of the two forms of heat, Dr. Whitelegge made the following experiment: He arranged an electric thermometer, which was set to ring at 212 deg. F., and placed it between 16 or more layers of blanket. In a steam apparatus the maximum interval of heat penetration indicated by ringing the bell was 17 minutes; whilst in a hot-air chamber with 12 layers of blanket a penetration temperature of only 196 deg. F. was reached after eight hours' interval, although the inlet temperature was 255 deg. F. and the outlet temperature ranged between 245 deg. F. and 250 deg. F. The ways in which injury is done to articles are somewhat different in the case of steam and of dry heat; thus scorching or partial decomposition of organic substances by heat shows itself in early stages by change of colour and texture, and by weakening of strength, and occurs sooner in flannel, blankets, and other woollen materials than in cotton or linen, whilst horsehair can be exposed to a very high temperature without injury. Most materials will bear a temperature of 250 deg. F. without being seriously injured, but above that signs of injury appear. Flannel and blankets exposed to steam at 260 deg. F. for half an hour become yellow, and their textile strength is lessened. Exposed to dry heat of 220 deg. F. for four hours, or to steam at 228 deg. F. for half an hour, white flannel became slightly yellow, but its textile strength was not impaired. Wool, cotton, silk, and paper could be heated to 250 deg. F. without injury if only for three hours, but a temperature of 295 deg. F. for three hours definitely singes white wool, and less so grey and white cotton, white silk, and paper and linen, but does not greatly spoil their appearance. The same temperature continued for five hours both singes and spoils the appearance of all these materials. Scorching is specially likely to occur when the article is exposed to radiant heat; thus in one experiment it was laid on wooden bars immediately over the heated bottom of the chamber, and showed distinct stripes of scorching, corresponding to the interspaces between the bars, whereas a pillow exposed to a greater heat, but screened from direct access of heat rays, was quite uninjured.

Over-drying, but short of actual scorching, renders many organic substances brittle, and so injures them; but on exposure to moisture they recover. When materials have been injured by over-drying, they should remain long enough in the air to recover their normal moisture before they are manipulated.

A temperature of 212 deg. F., whether dry heat, steam, or boiling water, fixes many organic colouring matters, such as blood, fæcal matter, &c., and the stain cannot subsequently be removed; hence they should be removed with due precautions in cold or tepid water, and then the articles thoroughly disinfected by heat. Furniture cannot be disinfected by heat, as, if varnished, it will blister, and the effect of steam is to soften the glue and loosen the veneer. If, in disinfection by steam, woollen materials are much wetted, they shrink and felt very much; and coloured materials, in which the dye is not fast, will run. On the other hand, dry heat, short of actual scorching, has no effect on colours, and causes but little shrinking of woollen materials.

### Mental Nursing.

ALTHOUGH it is perhaps unnecessary that a mental nurse should know the many varying types of insanity, it is well that she should recognise certain broadly-defined varieties, and the best mode of treating them.

A little experience will soon teach her to discriminate between the brooding melancholiac, who must be roused and interested, and the restless, vivacious maniac, who must be soothed and quieted. She will learn to detect the intent, listening look of the victim of aural delusion, who must be kept under the strictest surveillance, and know that a little wholesome neglect is sometimes very salutary for morbidly hysterical patients, whose craving for notice and attention causes them to simulate the symptoms of other diseases. The present age of extreme civilisation has fostered a certain hyper-sensitiveness which, when allied to the neurotic temperament, produces a class, often refined and cultured to a degree, subject to hysterical insanity, any severe strain causing the balance to tremble of those unstable brains. Such patients rarely describe their symptoms without exaggeration, so that a great deal depends upon the observation and veracity of the nurse, who must be able to note the effect of the different drugs, bromides, tonics, cannibis indica, and the various anodynes and soporifics which are so extensively used. It is an authenticated fact that to the foregoing class belong those appallingly frequent cases of puerperal insanity which form so large a proportion of the female patients in asylums, and it is a step in the right direction that the mental nurse is now expected to have physical training as well, with some knowledge of obstetrics, and it is desirable that the nurse should understand the use of the nostril and œsophageal tubes, for there is often a perverted taste for injurious matter, with an absolute disrelish for nourishing food, a marked feature of mental aberration.

Of late years there has been a great change in the constituents of food of our asylums. There is now less of the proteid material which is stimulating. Meat is seldom desirable, and beef-tea is rarely ordered except for senile decay or paralysis, whilst in maniacal cases it is decidedly injurious. Eggs and milk form an ideal food for the insane, for to fatten the neurotic patients is to practically cure them; especially is this the case in those excitable, voluble patients who talk, laugh, or sing night and day, taking little if any rest or food. But the physical nurse must recollect that the pathological condition of the insane differs considerably from that of patients whose nervous system is under control; especially is this seen in temperature and pulse.

Among the epileptics, the largely predominating class as regards number in our asylums, the rise and fall of temperature is one of the most reliable indications of an approaching seizure, and in those whose mental state may be due to decay or disease of the medulla, the pulse is often curiously affected, being very slow, with long lapses between its beats. Another test of the physical state of the patient is the nature and character of delusions, for very often the most frequent of these are due to some bodily ailment. Patients will often complain of rats gnawing them, or of wheels turning within their heads, of creeping sensations; and often some organic disease may account for those symptoms which, in themselves, are not wholly imaginary, though the patients ascribe their suffering to imaginary causes. It is this thoughtful study of their mental peculiarities which makes the true nurse, for anyone who puts down their whimsicalities to "pure cussedness" will naturally resent their conduct, when really it is as purely involuntary as the high temperature of pyrexia.

The most dangerous patients are those whose mania is homicidal or suicidal. The most homicidal patients are epileptics and victims of aural hallucination, but during the severe mania of puerperal insanity (which, happily, is often of short duration) the poor woman, frequently a loving and devoted mother, may in some unguarded moment become a murderess. Suicidal patients are rarely homicidal, and *vice versâ.* Suicidal mania is often found in those suffering from religio-mania, and in those victims of delusions of great fear, who believe themselves in the power of enemies. Paradoxical as it may seem, patients who harp continually on their lost condition and the wrath awaiting them will seize any opportunity to attempt to kill themselves. But the patients who threaten suicide loudly are rarely the most dangerous in this respect, for this form of mania is often latent, whilst homicidal madness rarely is. When it is remembered that the habit of uttering those dramatic threats may have been formed to frighten or provoke their unhappy relatives (many of whom are themselves of excessively nervous temperament) it is easy to understand the salutary effect of the asylum *régime.* Those patients rarely come until their friends have tried every possible device, wise or unwise, for their welfare, and when one realises what a trying charge a lunatic is in a private house one can only pity their predicament.

Very unwisely do relations often deal with those unfortunate patients, alternately yielding to their most freakish notions, or trying to restrain them in a way which would certainly be illegal in any well regulated asylum, so that separation from a jarring, irritated family is often the first step towards their recovery; but no wise nurse will allow such patients to harp on family grievances, or talk about delusions, even should they only be some of those amusing imaginings of personal grandeur so often exhibited by hysterical women. They should not be addressed as countess, duchess, or any other fictitious title they may have chosen for themselves, nor should they be allowed to wear any crown or insignia of imaginary royalty, for this is a relic of the good old days when the unfortunate denizens of Colney Hatch or Hanwell were exhibited to morbid sightseers for a consideration. Besides the difficulty of feeding, there is that of dressing them. Many patients persistently rend and destroy everything, therefore it is necessary to dress them in untearable canvas. Whenever possible, however, they should be permitted to return to everyday dress. This is more especially the case if the patient be a woman, for it is infinitely better that the nurse should keep strict watch over nimble, restless fingers than that she should feel the degradation of wearing a hideous, leather-like garment a moment longer than is absolutely necessary.

Many nurses are ignorant of the bearing of our lunacy laws in regard to the incarceration of patients in lunatic asylums. According to Act of Parliament (Vic. 95), it is illegal to use any mechanical restraint between the hours of ten a.m. and six p.m. Patients may not be locked into any room, nor tied into bed, nor subjected to any mechanical restraint. For instance, in hospitals a sheet is often wound round delirious patients and secured with safety-pins. To do this in an asylum would involve the medical superintendent in a serious breach of discipline; nor may locked gloves or boots be worn without being duly entered in the books of the asylum. Another difficulty is to provide suitable recreation for the patients. There are many who can scarcely follow the simple figures of a quadrille; but most mental nurses are expected to be musical, or, at least, singers, and if a gay, catchy tune be played they will enjoy Swedish exercises or a simple children's game, and the marching and countermarching to gay music will while away the long hours of a winter afternoon. Whatever the recreation be, let it be of such a nature as shall lift the patient out of brooding over the chimeras of a diseased brain, for the mental attitude of the insane is a moody introspectiveness, from which they must be won by some light task or agreeable occupation. Of course, a mental nurse will see that no scissors are given for needlework, &c., and in any outdoor sports a golf club or tennis racket might prove a formidable weapon in the hands of any irresponsible person. It would be wrong to deny that there is an element of danger in the work, and this renders it suitable only to such as are of good physique, and anyone at all subject to depression or irritability should certainly not think of taking up what demands strong nervous balance and stability.

### Training in the Provinces.

(*Continued from page* 31.)

### WARNEFORD HOSPITAL, LEAMINGTON (113 BEDS).

### TERMS OF TRAINING.

Candidates for training in the Warneford Hospital must be well educated and not under 22 years of age. If engaged after a month's trial, they are required to sign an agreement to remain in the service of the hospital for three years from date of entry, and the agreement includes a clause restraining them from acting "as a nurse for remuneration," either on their own account, or in connection with any institution within six miles of Leamington parish church during a further period of three years after the expiration of that time, under a penalty of £20. If a nurse leaves the services of the hospital before her three years' engagement is at an end she forfeits £6, and is expected to give three months' notice of her intention. No salary is given for the first year; for the second, third, and fourth years salary rises from £15 to £20, and then to £25 per annum, increasing up to £30. At the end of the first year probationers, if satisfactory, are promoted to the rank of assistant nurses, working in the wards of the hospital for the second year, and joining the private nursing staff in the third year, at the discretion of the matron.

After finishing their training, if retained in the service of the hospital, nurses' salaries rise by £5 a year to £30, sisters receiving £30 per annum, and in addition £5 for uniform. Probationary nurses are provided with uniform, consisting of two print dresses, three caps, and eight aprons, which they are required to wear on or off duty. Outdoor uniform is supplied to first-year probationers only. Certificates are given at the end of the three years' engagement to nurses who have passed satisfactorily through their training. Promotion to a post as sister depends on general suitability. No nurse is eligible for such promotion with less than three years' training. Night duty is taken by nurses in their second year, not more than six months altogether, usually for three at a time. Nurses who wish to join the Royal National Pension Fund are assisted to do so by the hospital authorities.

### HOURS ON AND OFF DUTY.

Nurses go to their wards at 7 a.m., leaving them at 8.30 p.m. During these hours they are given two hours daily for recreation, half an hour each for dinner and tea, and half an hour during the morning for luncheon, &c. Nurses and probationers are off each day from 2 to 4 p.m., or from 5 to 7, taking these times alternately. On Sundays they are off duty either from 10 a.m. to 1 p.m. or from 4.30 p.m. to 9.30 p.m. A day off is allowed once a month from 9.30 a.m., and twenty-one days' leave of absence is given annually. Nurses and probationers who are off duty are expected to attend the hospital chapel at 3.15 on Sundays, and 5.30 on Thursdays.

### MEALS.

All meals are served in the nurses' dining-room, and there are no food allowances except to night nurses. The day staff breakfast at 6.40 a.m.; dinner is at 12 30 and at 1 p.m.; tea at 4 and at 4.30.; and supper at 8 and 8.30 p.m.

### LEICESTER INFIRMARY (240 BEDS).

### TERMS OF TRAINING.

Three years' training is offered to women at the Leicester Infirmary. Candidates should be between 23 and 35 years of age, and must be not under 5 feet 3 inches in height, according to the regulations. Applicants are required to pass a preliminary examination in elementary anatomy, physiology, and sick-room cookery, before being admitted upon a month's trial. These tests satisfactorily undergone, a three years' engagement is entered upon, with a certificate and a grant of £8 at the conclusion of the time. Salary commences at £8 the first year, rising to £14 the second, and to £16 the third. Laundry and in and out-door uniform are provided by the infirmary. A limited number of "lady probationers" are also received for three years' training on terms similar to those already given, except that a premium of £15 is required from them either on signing the agreement or in three sums of £5 each within the first year; or the sum may be deducted from their salaries during the three years. Salary is for the first year £12, for the second £16, and for the third £18. A few "lady pupils" are received for six or twevle months' training, paying £10 10s. a quarter, and defraying their own laundry expenses. They are guaranteed a certificate on completing one year's training.

Nurses who are appointed on the permanent staff after finishing their three years are paid salaries ranging from £22 to £32 per annum. Sisters, who must have had at least three years' training, receive from £26 to £40 per annum. Night duty is taken for ten weeks at a time in alternation with twenty weeks' day duty. From a fortnight to a month's annual holiday is given.

### HOURS ON AND OFF DUTY.

Day nurses are on duty from 8 a.m. to 9.30 p.m., with time for meals and two hours off duty daily. A half-day is given once a month, from 1.30 to 9.30 p.m. On Sundays the hours off duty are from 10.30 to 1, 3.30 to 5, or 2 to 3, and 6 to 8.15.

Night nurses are on duty from 9.30 p.m. to 8.45 a.m., being free from 10 to 12.15 each morning.

### MEALS.

Day nurses' meals are as follows: Breakfast, 7.30 a.m.; lunch, which is served in the ward kitchen, between 10 and 12; dinner, 1 or 1.30; tea, 5 or 5.30; and supper, 8 or 8.30. Tea and all other meals, except the early lunch, are served in the nurses' dining-room. No weekly stores of butter, sugar, &c., are given out to the nurses.

### Wants and Workers.

[THE attention of correspondents is directed to the fact that "Helps in Sickness and to Health" (Scientific Press, 28 & 29, Southampton Street, Strand, London, W.C.) will enable them promptly to find the most suitable accommodation for difficult or special cases.]

NURSE E. R., Castle Hill, Cricklade, Wilts, wishes to hear of a home for the winter months for nurse with weak throat and chest. It should be inland, dry, and sunny. Terms, a small sum weekly.

A. C., 29, HILLFIELD AVENUE, Hornsey, would be glad to know of a good home in suitable neighbourhood for case where she could place her invalid mother for the winter; chronic bronchitis and weak heart. Good living essential. Terms must be strictly moderate.

### Nursing in Paris Hospitals.

### C.—THE NURSING SISTERS.

### V.—Popular Estimation.

Victor Hugo, in one of his most striking chapters of "Les Misérables," tells us that the street arab of Paris, who respects nothing else, is always polite to Sisters of Charity. The fact is, the Paris *gamin* is the quintessence of the spirit of the city—cynical, critical, satirical, sceptical, but moved to something like respect and even reverence when real suffering encounters real self-sacrifice. The nursing Sisters have furnished Paris with more than one municipal saint, canonised in the popular mind, especially in the terrible cholera epidemics which Paris has gone through. Readers of Eugène Sue's masterly descriptive romances of the town will recall examples of this feeling.

It is evidence of how bitter have been the passions evoked by the political events of the last thirty years, and how deep-seated has been the popular tendency to attribute to the alleged clericalism of the Empress Eugénie the responsibility for the disasters of 1870 and 1871, that the laicisers ever dared attack the position of the nursing Sisters, and the fact that they have so nearly succeeded in completely evicting them. Almost anyone before 1870 would have laughed to scorn the idea that innovators of any sort would be tolerated in an attack on the nursing Sisters, still less be able to accomplish their ends. It seemed a matter of course to the humble Parisian that in case of serious accident or illness he should be placed under the charge of a Sister of Charity. Evidence of this is found in one of the curious results of the recent laicisation mentioned by M. Ferdinand Duval in the great debate on laicisation in the Municipal Council on December 24th, 1890. Said M. Duval, "We cannot help remembering that in our youth no religious order existed which nursed the sick in their homes; there were Sisters in the hospitals only. The inconvenience of the laic nurses has given rise to quite a literature. To-day, when a man attacked by any complaint is fortunate enough to be able to be nursed at home, without the pain of separation from his own folks when most he needs them, be he Catholic, Protestant, or Freethinker, what does he do? He sends for a Sister and a hospital doctor, and when the doctor arrives first, it is he who sends for the Sister. What we ask is, that what the rich man can have the poor man also shall be able to find in a hospital when he so desires it."

Anyone who to-day traverses the corridors and wards of the Hôtel Dieu or of Saint Louis, or the endless network of the old prison of Saint Lazare, where the Sisters still reign, cannot fail to be struck with the breath of peace and solace which a Sister diffuses to patients and prisoners as she goes about her duties. It is incontestable that the Parisian in trouble has been accustomed from time without mind to this spectacle, and it is a great wrench to his feelings to be deprived of it. In Article III. of this series I referred to the undoubted immorality of the laics as compared with the Sisters in the matter of honesty, sobriety, and especially of the levying of blackmail on patients. In this regard M. Maxime Du Camp, in his monumental work, "Paris," has some remarks so apposite in their bearing that I cannot refrain from quoting them in full. To fully appreciate this it must be borne in mind that the Du Camp work was partly issued before the beginning of the third Republic, and that these words were penned before any idea had been broached of increasing the lay element in the nursing staff at the expense of the religious portion.

Treating of the general nursing staff in the Paris hospitals, M. Du Camp explains that "the nuns are not sufficient to give the patients all the required attention. The assistance in the hospitals includes hired men and women, called second-class servants, who are, properly speaking, ordinary male and female sick nurses. The first number 491, the second 499. This is the defective side of the institution, and the administrative and scientific chiefs are unanimous in recognising that, save certain exceptions, this staff is deplorable. Recruited in a bad class of the population—among discharged workmen, servants out of place—they give no gratuitous service to patients, who must always have money in hand to soften hearts possessed more by venality than compassion."

These words seem to be an echo of, though really so long antecedent to, my first gleanings from hospital directors concerning the new laics as detailed in Series A of these articles. Neither in 1863 or in 1898 would any such character be given to the Sisters by friend or foe. M. Du Camp continues:—

"We must admit that to have all the qualities necessary for a good nurse one must be an angel, and that few men would be capable of fulfilling this very painful office. A nurse has at least ten beds to watch, and she is called upon to do the most repugnant tasks. How are they paid? They have, beside lodging, food, and uniform, a wage varying from 15 to 21 fr. [Note.—On an average each male nurse costs the administration 79 fr. 59 cents., and each female nurse 66 fr. 58 cents. per month.] It is very difficult to find paragons at this price; but it is the patient who has to pay, and not uncommonly a nurse makes 40 and 50 fr. a month in tips.

"Their great fault is drunkenness. No one knows how to keep wine out of their reach; at the Hôtel Dieu, at Lariboisière, the jugs which go back and forth from the cellar to the wards are padlocked, and the cellarman and the Sister only have keys: useless precaution, they know how to introduce into the best closed vessel a straw, or perhaps a catheter stolen from the doctor, and the ration is always short on arriving at its destination. They drink the quinine wine; in the lying-in wards the female nurses steal the rum used for reanimating the half-dead infants. Much more, the surgeons who make anatomical preparations have to double-lock them, because the male nurses have the ghastly courage to drink the alcohol in which the human remains are preserved. Moreover, the more I study this world of ignorance and misery, the more I come to the conviction that drunkenness is the cause of 80 per cent. of all the evils."

M. Maxime Du Camp then goes on to remark on the old, old complaint of lay nurses being mere temporary servants, taking up the work from lack of other resource, and seeking to leave it as soon as any chance offers, and also the recruitment among ex-patients, especially at Saint Louis, from disfigured persons. All this shows how long established have been these very evils I have before referred to. Finally, M. Du Camp remarks on the one shining virtue of the secondary laics under the old system—drunkards, degraded, dishonest, and avaricious as they were—that in times of greatest danger and panic, even in the most terrible cholera epidemic, they never deserted their posts.

Of course, no one would ever broach the question of a Sister deserting her post at such a time. The public mind is so accustomed to take this faithfulness as a matter of course that even M. Du Camp does not allude to it. What is startling, however, is the notion of this deplorable lay element in the old nursing system, instead of its being merely remedied, being expanded in a few years upwards to oust the very element which was the saving grace of the old system, and whose virtues were such a matter of common notoriety as not to need to occasion even a passing word when treating of the defects of their subordinates.

Of course, the unscrupulous enemies of the Sisters would undoubtedly lay on the religious element the responsibility for the defects of the secondary staff in the time of Maxime Du Camp's study. It would be a very unjustified idea. The hospital chiefs must take the whole responsibility for the character of the material they recruited as nurses. This is why their responsibility is so great in rejecting in bulk as nursing material the Sisterhoods which have stood the test of popular criticism for over a thousand years, and which survived the great violently anti-clerical outburst of 1789 for nearly a hundred years, because the popular will was so unanimous in regarding the nursing nun as the neutral object of universal esteem for all persons, of whatever creed or of no creed at all.

Edmund R. Spearman.

### Our American Letter.

There have been great changes in the *personnel* of the New York City Training School. In June of last year the Commissioners of Charity decided to put the nursing school and the hospital each on a separate basis, an arrangement that has proved very beneficial to the school. Later in the year the superintendent and assistant superintendent resigned the positions of chairman and secretary-treasurer of the School Co-operative Registry on account of the increasing pressure of work, which society has been incorporated with the Graduates' Co-operative Registry. Miss Darch last autumn inspected the Infants' Hospital in Randall's Island at the request of the authorities with a view to ascertaining what advantages it offered for the training of nurses, and in consequence so overtaxed her strength that she was obliged to send in her resignation. The same thing has happened to her successor, Miss Kimber, and her assistant for the previous two years, Miss Mary S. Gilmore, has been appointed to succeed her, with Miss Theodora Le Febvre as her assistant. The male and female departments have been separated because of the heavy work attached to the position, and Miss Amanda Silver, who has been supervising nurse of the male school, has been appointed superintendent of that department.

The number of nurses graduating in the female school was 25, and in the male 16. Ten of the male pupils went to the front during the recent hostilities. The school was fortunate in obtaining one of the five prize medals awarded at the Trained Nurse Educational Exhibit in April last. The subject chosen was a model room in a maternity hospital.

The real objection of the American Government to the employment of women nurses in the field was the difficulty of providing them with adequate accommodation. The Surgeon-General of the American army, however, having accepted the offer of the American Red Cross Society to provide camp hospitals and tent accommodation as well as transportation, a plan for placing additional nurses in the army hospitals is being carried out by Dr. Anita McGee, in conjunction with Mrs. Winthrop Cowdin, acting president of the Red Cross Auxiliary for Maintenance of Trained Nurses in Army Hospitals. All accepted nurses must be certificated and come up to a fixed standard, when they will be officially enrolled. Both Dr. and Mrs. Lesser, who represented the Red Cross in Cuba, contracted malarial fever and have been seriously ill.

Miss Odou, who was trained at St. Bartholomew's and Queen Charlotte's Hospitals, London, and at Vienna College, and who is known in New York by her district work in connection with the Floating Hospital and St. John's Guild, is establishing a training school for embalming the dead! She deems that the care of the dead, and especially of dead women and children, lies within a woman's sphere of work.

Eighteen volunteer nurses, who decline to permit their names to published, undertook the care of the sick of the First Illinois Cavalry at the Post Hospital, Fort Sheridan. They gave their services gratis, and were not at all overjoyed when, after three weeks' work, they heard that Surgeon-General Sternberg had decreed that all volunteer nurses were to be paid at the rate of 30 dols.—or £6—a month. The rate of remuneration is not large, but they feel that, having volunteered, any payment detracts from the status of their work.

### Appointments.

Darenth Schools for Imbecile Children.—On the 11th inst. Miss Alice Skelton was appointed Superintendent Nurse of these schools. She was probationer, staff and charge nurse successively at her training school, St. Thomas's Hospital, and has since been charge nurse for three years at the North-Eastern Fever Hospital, and night superintendent at St. Pancras Infirmary.

Warwick Union Infirmary.—Nurse Charles, who for the last five years has been charge nurse and trainer of probationers at this infirmary, was on October 15th elected Superintendent Nurse. She was trained at the Chorlton Union Hospitals, Manchester, and has been, at different times, charge nurse Union Infirmary, Leeds, and charge and maternity-nurse at the Doncaster Union Infirmary.

Hospital for Women and Children, Savannah, Georgia.—The new Matron of this hospital is Miss Eleanor Wimbush. She was trained at the Hull Royal Infirmary.

### Minor Appointments.

Hospital for Women and Children, Savannah, Georgia.—Miss Clara Eckles, who was trained at, and has been on the staff of the Hull Royal Infirmary, for the last nine years, has been appointed Sister here, and sails with the new matron to take up her new appointment.

### Mediæval Practice in the Care of the Sick.

### X.—THE QUESTION OF DIET.

ANOTHER subject which comes within the province of a nurse is the diet of the sick and convalescent.

The food recommended by physicians varied according to the patient's "complexion." One writer finds fault with doctors who in their experiments do not take the peculiarities of each patient into consideration. As a rule, wounded men were required to abstain from wine at the beginning of their sickness; barley water, chicken broth, soup "ootes with almaunde mylk," and such kindred forms of nourishment, were given to people really ill. "Small chickenes and of smale briddies and kidis and lambis and calves," with "Wiyn of pomegarnatis," for those needing something more substantial. Beef, goose, "all manner of mete that is maad of sweet paste," milk, water, applis, peris," and new wine, are placed upon the index of forbidden things for patients liable to stone, but "pork" and fish that have "schellis" are allowed.

Mutton was considered a most valuable article of diet; in Lydgate's "Horse, Sheep, and Goose," printed by the Early English Text Society, we read that sheep's "flesche . . . is naturalle Resturacion, . . . affter with grete syknes. Rosted or so done holsum is mutton. 'Boylyd . . . fulle nutrityffe after a great excesse.'" Sick people were sometimes wrapped in the sheep skin of an animal freshly slaughtered, and from a sheep's "hede boyled hole and alle, there cometh a jelly, an oynment full royal; for ache of bonys and also for brasue hit remedyethe and dothe ease, belyve." "Black shepys wole with freshe oyle and olyve at a strayte nede, can well stanche bloode."

In a fable on "Li Sainereffe" we find that women were employed to perform the operation of bleeding, practised in old times upon all persons alike, well or ill, generally at the change of the seasons. In the "Ancren Ruele," written during the early part of the 13th century, Bishop Poor orders the ladies to "have your hair cut four times a year to disburden your head, and be let blood as oft, or oftener if it be necessary." He forbids them to do much beyond amuse themselves for three days after the blood letting; while a book, which probably once belonged to the Abbey at Glastonbury, advises that patients should not be bled for "fifteen nights ere Lammas or after it for five and thirty nights, since then all venemous things fly and much injure men." Qualified physicians rather resented the interference of women in their science. "Lanfrank's Science of Cirurgie," written in the 14th century, contains a complaint that blood-letting is left to "barbours and women."

The charms and white witchcraft practised by harmless wise women, though interesting enough, hardly belongs to the subject of nursing the sick, but the "Saxonia Scriptures," written about 1040, contains magic charms and cures for elfin diseases, "over looked people" and "devil sick" folk, as well as affording terrible suggestions of the suffering entailed by women's diseases in those ignorant days. Holy water mixed with spring wort figures in a recipe for typhus fever; and dust from places where Saint Mildred had put her foot would "cure sundrie diseases" if "dronken," but doubtless faith was a large ingredient in such cures. The pious, who had masses said over their medicines, and the nurses, who measured time in the preparation of their simples by reciting Pater nosters, aves, and Credos, cannot with justice be called superstitious; but all "exorcism of fever," in which the "sacramental paten," "holy water," the last gospel in the mass, and the psalm "Beati immaculate" are used as part of an incantation, cannot, one would think, have received the sanction of the Church.

That the remedies employed were frequently far worse than the diseases may well be imagined; we will give a single instance. Who would not prefer any amount of "heartburn" to swallowing "earth gall and pepper in warm water, three bowls full, at night fasting?" and some of the treatment which belong to midwifery, rather than general nursing, was so barbarous that one hardly wonders the author of "Holi Maidenhood" should wax eloquent upon "the old wives' indelicate skill" in extolling the advantages of a celibate life.

What modern nurse has not endured a martyrdom at the hands of a patient's injudicious but kindly friends? We will conclude this imperfect review of mediæval nursing with an account of a well-meaning woman's visit to a sick friend, which has been preserved in the evidence of a witness in a sixteenth century libel trial. "This respondent sais that 'upon a certain tyme Constance Wade was sick, and this respondent, as one neybour would do to another, did bring her chickens, spices, and other hings to comfort her; and after that hit chanced to the respondent to be sick, and the said Constance brought this respondent a little quantity of beste' (rich thick milk of the cow directly after calving), to relieve her mouth, and said, 'Bie her troth! she was ashamed that she had not some better thing to bring her!' Also at the same time she brought a little salt eele with her." If "this respondent" was not too ill to loathe all food, and if she indulged a weakness much to the taste of Lady Jane Ingoldsby, her nurse must have required tact to dispose of the beste and the salt eeles without injury to the patient.

### A West London Maternity Home,

### 87, FULHAM ROAD.

So often are we asked by trained nurses if there is any institution where they may be trained for the certificate of the London Obstetrical Society on reciprocal terms, that an account of a private home in which this can be done may be useful. It is situated in the comparatively open district of Fulham, surrounded by a rapidly increasing neighbourhood, inhabited chiefly by artisan and labouring classes. It is only a small place at present, but the work has grown so enormously that in the spring the owner will remove to a large and convenient house which she is having built for the purpose. She is a certificated midwife, having studied at the St. Barnabas in East London, at the London Hospital for a couple of months, and finally at the British Lying-in Hospital, Endell Street. Before beginning work on her own account, she had some experience of district work in the parish of St. Clements, Fulham. The home receives one or two patients, and as many pupils as can be accommodated; it is comfortably furnished and well kept. Two classes of pupils are taken, the fully-trained nurse, who for her training for the L.O.S. gives her services for six months, and the non-professional pupil who desires to become a monthly nurse, and who pays £15 15s. for board, residence, and instruction for three months. All see plenty of cases, and Nurse Heatley, the organiser and owner, gives a weekly lecture on the theoretical part of the work, whilst a resident doctor in the vicinity gives another. Nurse Heatley charges her patients according to their means; when anyone can afford to pay £1 1s. she will not nurse them for less. In the event of medical aid being required, she shares this fee with the doctor called in, and is responsible that he is paid. The majority pay, however, smaller sums, and are responsible for their own fees should a doctor be needed; nevertheless, Nurse Heatley collects it for him, often in small instalments.

### Everybody's Opinion.

[Correspondence on all subjects is invited, but we cannot in any way be responsible for the opinions expressed by our correspondents. No communication can be entertained if the name and address of the correspondent is not given, as a guarantee of good faith but not necessarily for publication, or unless one side of the paper only is written on.]

### WINTER HEALTH RESORTS.

MISS HENRIETTA DENDY writes: In your latest edition of THE HOSPITAL, in an article on "Winter Health Resorts," may be found the following words: "Madeira and the Canaries are useful in febrile irritability and dry cough, but they are both somewhat relaxing." If you can find space in your next number, I should like much to enter a protest against this statement as regards Grand Canary. I have spent three winters and one summer at Las Palmas in the double capacity of patient and nurse, and can therefore speak with authority derived from personal experience. Except on the very rare occasions when a south wind is blowing, the climate of Las Palmas is dry, warm, and invigorating. The latter quality arises from the prevailing north-west wind, combined with the exhilarating bright sunshine. I consider the climate an ideal one for persons with a rheumatic tendency or in the early stages of phthisis. Four years ago I was declared by a consulting physician unable to spend another winter in England, on account of the critical condition of my lungs (incipient phthisis). I had tried Bournemouth without any good result; the Canaries were suggested as a possible living place for me. I am now so well that the same physician sanctions my spending this winter in England. Therefore I am grateful for the Canaries, and shall be glad if the knowledge of my experience may lead others to benefit also. I enclose my card, and shall be glad to supply any practical details as to voyage, accommodation, &c., to anyone desirous of having them.

### THE NURSE'S BICYCLE.

THE SUPERINTENDENT OF A NURSES' CO-OPERATION writes: We have heard a good deal about the sins and wickednesses of private nurses one way and another, and it seems to me their latest fault is their taking their bicycles with them when they go to a case; and as it is generally impossible for a nurse to know whether this will be convenient or desirable, such an act shows, to say the least of it, a want of the first principles of good manners. Now we all know there is no reason whatever in many cases why a nurse should not have her bicycle with her and thoroughly enjoy good rides when she has the chance; also, judging by the number of bicycles that are given to nurses by grateful patients or their friends, I am sure many are wishful for them to have this pleasure. When I first heard about this being a difficulty I could not help wondering whose fault it was, for I take it that a nurse does not go to a case without being sent by someone; and, therefore, the sender is responsible. I am glad to say we have had no trouble in this way in our co-operation, but our reference committee have felt it best to pass a rule that a nurse shall not take her bicycle to a case unless a written request to that effect has been sent to me by those employing her. We find this arrangement works very well, and I think it is one that may be found useful to other nursing associations, as under it nurses are not deprived of the pleasure of riding where circumstances permit it, and run no risk of arriving with their machines when it would be not only very inconvenient but out of place.

### THE POOR LAW NURSE.—WHERE THE SHOE PINCHES.

ALICE PETITT writes: I was greatly interested in an article appearing in THE HOSPITAL for last week concerning workhouse nursing, and I thought I should like to tell you one or two things that at present do not seem to be very much thought about. When the new rule came into force regarding nursing it did not exclude untrained women from entering Poor Law institutions as assistants; nor did it seem to enter the minds of the officials how unpleasant it would be for trained nurses to have to work with untrained so-called nurses. As the thing is getting more general, owing, I suppose, to the scarcity of nurses (but really making it far more difficult to obtain them), it would be a boon if someone would take the problem up and work it out. I worked with an untrained nurse for over two years, so I am writing from experience. The greatest difficulty in getting nurses seems to be in places where there are only three or four on the staff. It may be surprising, but in that number one or more untrained women will often be found, so that there are other evils besides being under the control of the master and matron that a workhouse nurse has to put up with. Then, again, why should an untrained nurse go in and take the same salary as a trained nurse, who has had to give time or money to gain her knowledge? There is also another thing which is very hurtful to a nurse's feelings—to say the least of it—and that is, the guardians or visitors take complaints made by the patients to the master or matron without first acquainting the nurse with the facts; complaints which, in my experience, are very often groundless. I have known a good many workhouse nurses, and I may say that the greater number of them entered into the work fully understanding the difficulties, but ready to do their best; but, meeting with nothing but discouragement, they at last gave up in despair. Nurses are supposed to put up with the same restrictions as in their probationer days, with less time off duty, owing to the workhouses being so understaffed, for if one nurse leaves, or takes a holiday, those remaining have to do double duty. Yet a nurse must always look cheerful and bright, although the work is of a most discouraging kind. They cannot hope to see many of their patients recover, knowing, as they do, that the greater number only come in to die or to live out years of suffering, which makes them fretful and hard to nurse. I don't think that even the Nightingale type of nurse could do it for long, though some of them have been in the field and some are still there struggling to do their best. If you will kindly read this paper, I am sure you will agree with me that the workhouse nursing question wants thoroughly working out. Do you think if correspondence were invited on the subject, and the letters laid before the Local Government Board, it would do any good?

*** The subject is certainly one that requires thoroughly threshing out, and a wide correspondence amongst and from every class of nurse working under the Poor Law could not fail to be helpful to those trying to solve the difficulties.—ED. *T. H.*

### Where to Go.

LECTURES ON MEDICAL RELIEF.—A course of eight lectures on "Medical Relief" will be given on successive Fridays at the Portman Rooms, Baker Street, at half-past four p.m. The first, which is entitled "The Growth of Medical Charities," will be delivered on the 21st inst. by Mr. C. S. Loch, and Sir William Broadbent, M.D., will deliver the introductory address. On the 28th inst. Dr. L. A. Hawkes will lecture on "The Dispensary," and he will be followed by Mr. Clinton Dent on "The Hospital," Dr. T. D. Savill on "The Poor Law Infirmary," Dr. Rayner on "The Lunatic Asylum," Miss Amy Hughes on "District Nursing," Mr. F. G. P. Neison on "Sick Insurance," and Dr. J. B. Hurry on "Self-supporting Dispensary and District Nursing Association." All particulars about the lectures can be obtained from the Hon. Secretary, Mrs. G. F. Hill, 19, Park Mansions, Battersea Park.

### For Reading to the Sick.

"THE Lord shall give thee rest from thy fear."

**Verses.**

Is God for me? I fear not, though all against me rise!
When I call on Christ, my Saviour, the host of evil flies.
My Friend, the Lord Almighty, and He who loves me—God!
What enemy shall harm me, though coming as a flood?
—*Paul Gerhardt.*

Theirs was the sin to cumber Faith with Fear—
To tremble—where they should have feared and loved;
To overlook the Glory, close and near,
And only reverence it in space removed.
—*Houghton.*

If the way be drear,
If the foe be near,
Let not faithless fears o'ertake us!
Let not faith and hope forsake us!
For, through many a foe,
To our home we go.
—*Zinzendorf.*

Why fear the Night? Why shrink from Death,
That phantom wan?
There is nothing in Heaven or Earth beneath
Save God and man.
But never for this, never for this
Was Thy being lent!
For the Craven's fear is but selfishness—
Like his merriment.
Know well, my soul, God's hand controls
Whate'er thou fearest;
Round Him in calmest music rolls
Whate'er thou hearest.
—*Whittier.*

In Heavenly sunlight live no shade of fear;
The soul there—busy or at rest—hath peace;
And music floweth from the various world.
—*Allingham.*

**Reading.**

FEAR NOT.

There need be no difficulty in distinguishing between the holy and blessed "fear of the Lord," which is our "treasure," and which is only as the sacred shadow cast by the brightest light of love and joy, and the fear which hath "torment" and is cast out by perfect love and simple trust.

Fear Him, ye saints, and you will then
Have nothing else to fear.

Precisely expresses the distinction . . . . Would our King tell us again and again, "Fear not!" if there was any reason at all to fear? Would He say this kind word again and again, ringing changes as of the bells of heaven upon it, only to mock us, if he knew all the time that we could not possibly help fearing? . . . . He says we are not to fear; see how we are to fear nothing, and no one, and never, and nowhere; see how He Himself is in every case the foundation and the grand reason of His Command, His presence, and His power always behind it; and then shall we hesitate to say, "I will fear no evil, for Thou art with me." The Lord is my light and my salvation; whom shall I fear? The Lord is the strength of my life; of whom shall I be afraid?

There is a "fear not" for every possible case and kind of fear; so that we have never any answer to give when He asks, "Why are ye fearful?" but we are without excuse. It is part of His "precious promises" that "thou shalt be steadfast and shalt not fear." It is one of the blessed results of His reign that His flock "shall fear no more." It is no impossible thing, but the simple and natural consequence of really seeking and really trusting the Lord that He will deliver us not from some, but from "all" our fears.—*From "Royal Commandments."*

### Notes and Queries.

**The contents of the Editor's Letter-box have now reached such unwieldy proportions that it has become necessary to establish a hard and fast rule regarding Answers to Correspondents. In future, all questions requiring replies will continue to be answered in this column without any fee. If an answer is required by letter, a fee of half-a-crown must be enclosed with the note containing the enquiry. We are always pleased to help our numerous correspondents to the fullest extent, and we can trust them to sympathise in the overwhelming amount of writing which makes the new rules a necessity.**

**Every communication must be accompanied by the writer's name and address, otherwise it will receive no attention.**

*District Night Nursing.*

(34) Can you give me any information as to the best way of arranging for night nursing in connection with district work?—*H. K.*

We believe every home and association has its own arrangements suitable to the local needs, and providing hours for meals and sleep for the nurses. You had best write to the principal district nurse associations asking them for their rules, stating why you want them.

*Book on Hospitals.*

(35) Kindly tell me the title of a book published by Sir Henry Burdett, the price of which, I believe, is 5s., and is issued yearly. The first part dealt with the expenses of different hospitals, the cost per bed or cot, &c. The second part gave a list of hospitals, giving income, committee, medical staff, name of matron, number of nursing staff, number of beds, special features. The third part contained similar details of children's homes, blind asylums, &c. Will you tell me whether this book gives the fullest details of hospitals as training schools for nurses, or whether Sir H. Burdett has published another.—*Sweep.*

You evidently mean "Burdett's Hospitals and Charities," published annually. If you wish a book solely for nursing particulars you will find "Burdett's Official Nursing Directory" meet your requirements. Both are published by The Scientific Press, 28, Southampton Street, Strand, W.C.

*Holt-Ockley System.*

(36) To whom should I apply for particulars of the Holt Ockley system of nursing?—*Douglas.*

To the hon. secretary, Mrs. Henry Lee Steere, The Cottage, Ockley.

*Training for Nursing.*

(37) I am 15 years of age, and anxious to train as a nurse. Could you advise me how to start gaining knowledge? Is there a St. John Ambulance Society where I could attend evening lectures; or perhaps there are other institutions?—*M. L. B.*

You are much too young to train as a nurse, but there is no reason why you should not attend ambulance classes. Write to the secretary of the Hospital Saturday Fund (Ambulance Branch), 54, Gray's Inn Road, W.C., for particulars of their lectures. You might also study books on nursing. Write for a catalogue to the Scientific Press, 28 & 29, Southampton Street, Strand, London, W.C.

*Probationers with Salary.*

(38) Are there any vacancies for probationers with salary the first year?—*E. B.*

Most hospitals pay first year probationers a salary. Get "How to Become a Nurse," by Honnor Morten, published by the Scientific Press, 28 & 29, Southampton Street, Strand, London, W.C., price 2s. 6d.

*Compulsory Testimonial.*

(39) Can a nurse demand a testimonial from her matron upon leaving a hospital to take up another appointment?—*E. M. S.*

Certainly not; a matron can always withhold a testimonial if she thinks fit.

*Marking Linen.*

(40) What is the best ink for marking hospital linen?—*Matron.*

There are many. Bond's is an old-established one, and a rubber stamp is given with it, if required.

*Elementary Training.*

(41) A young lady of 19, of small means, not intending to be a nurse, would like a little insight into simple nursing.—*St. Neots.*

See our advertisement columns.

*Monthly Fees.*

(42) I was engaged to attend a case from October 20th, but was called in on September 20th. I stayed a fortnight, but was obliged to leave through illness. I have now some difficulty in getting my fee for the time I was there. (2) Can I claim the fee from October 20th, as well as that due for the fortnight's nursing?—*Nurse C.*

You are, of course, entitled to payment for the fortnight's work. As you left, however, for your own convenience, you cannot claim anything more. (2) As you were able to attend the case at the earlier date, you cannot ask for payment at the later one.

*Lesson in Electrolysis.*

(43) Where are lessons given in electrolysis for the removal of superfluous hairs?—*M. A. R.*

We cannot recommend anyone. Electrolysis being a surgical operation, it ought to be performed by a surgeon.

*The Menopause.*

(44 Kindly tell me the name and publisher of a good book on "The change of life in women." Something inexpensive and simple is required.—*X.*

On the medical aspects of the question, "The Menopause and its Disorders," by Keith Napier. Published by The Scientific Press, 28 & 29, Southampton Street, Strand, London, W.C.

*London Obstetrical Society.*

(45) We regret that in our issue of October 1st, under this column, the address of the London Obstetrical Society was incorrectly given. It should have been 20, Hanover Square, W.

